# A Study on the Evaluation of Ultrasonic Propagation Properties and Nonlinearity According to Temperature Changes of Aluminium Alloys for Each Aluminium Alloy by Temperature

**DOI:** 10.3390/s25247494

**Published:** 2025-12-09

**Authors:** Junpil Park, Jaesun Lee

**Affiliations:** 1 Extreme Environment Design and Manufacturing Engineering, Changwon National University, Changwon 51140, Republic of Korea; junpil@changwon.ac.kr; 2 School of Mechanical Engineering, Changwon National University, Changwon 51140, Republic of Korea

**Keywords:** ultrasonic testing, wave velocity, nonlinearity, aluminium alloy, temperature measurement

## Abstract

Aluminium alloys are widely used across various industrial sectors due to their suitability for enhancing structural safety and reducing weight, thereby improving operational efficiency. This study investigates the feasibility of using ultrasonic techniques as an alternative to thermistors for temperature monitoring in electric vehicle motors and batteries. The extent to which ultrasonic maximum amplitude and propagation velocity are temperature-dependent was examined, and the material nonlinearity was analyzed. Step-wedge specimens of Al3003, Al6061, and Al6063—commonly used in electric vehicle components—were fabricated with thicknesses of 4, 6, 8, 10, and 12 mm to examine thickness-dependent behavior. Although the three alloys differ in composition and mechanical properties, their ultrasonic propagation characteristics were found to be highly similar. As temperature increased, ultrasonic attenuation increased while propagation velocity decreased. For intact specimens, nonlinearity increased with temperature. However, the variation remained constant beyond a certain temperature range. In contrast, tensile-fatigued specimens showed increased nonlinearity with fatigue cycles, and the variation decreased at elevated temperatures, producing a more pronounced nonlinear response. These findings suggest that ultrasonic techniques may provide a cost-effective solution for temperature measurement and defect diagnosis, potentially replacing high-cost thermistors currently used in electric vehicles.

## 1. Introduction

Global efforts to mitigate climate change have accelerated the transition from conventional internal combustion engine vehicles to electric vehicles (EVs). In EVs, the battery pack serves as the primary energy storage system. However, its substantial mass—typically 400–500 kg increases the overall vehicle weight and reduces driving range. To address this issue, ongoing research has focused on structural optimization and lightweight materials. A 5% reduction in the mass of a 1500 kg vehicle, for example, can improve fuel efficiency by approximately 1.5% and enhance power performance by 4.5% [1]. Despite these advantages, the safety of EV batteries has emerged as a major concern, as thermal runaway–induced fires can lead to severe property damage and human casualties. Consequently, reliable temperature monitoring and concurrent defect detection have become increasingly important.

Aluminium alloys are widely applied in automotive components such as chassis, suspensions, battery housings, motor casings, and refrigerant manifolds due to their light weight and suitable mechanical properties. However, during manufacturing processes such as casting, forging, and machining, defects including pores and micro-cracks may develop. Additionally, the relatively low strength and softness of aluminium alloys may contribute to processing-related defects. Because aluminium casting is especially prone to pore formation, extensive research has been conducted on improving aluminium moulding processes in both academia and industry [2,3]. To overcome the intrinsic weaknesses of pure aluminium, alloying elements are added to enhance mechanical properties such as strength and corrosion resistance. Aluminium alloys of different series exhibit variations in mechanical behaviour depending on alloying elements and manufacturing processes. Most aluminium components used in vehicles have thicknesses of 4 mm or more, with some reaching up to 12 mm to ensure sufficient strength and effective cooling.

For components requiring thermal management—such as EV batteries and motors—temperatures are typically monitored using high-precision sensors such as thermistors, which maintain operation within a safe temperature range. Although thermistors offer excellent accuracy, they increase manufacturing costs and may introduce failure risks such as self-heating or short-circuiting. Therefore, this study aims to replace conventional thermistors with a nondestructive ultrasonic technique capable of both temperature monitoring and defect detection [4,5,6,7,8,9].

Ultrasonic nondestructive testing is widely used to detect internal defects such as pores and cracks in various industrial materials. It is well established that ultrasonic propagation characteristics, including attenuation and velocity, vary with temperature [10,11,12,13,14]. Since these propagation characteristics are also influenced by internal defects, this study analyzes the temperature-dependent ultrasonic behaviour of three aluminium alloys commonly used in EV components: Al3003, Al6061, and Al6063. By measuring temperature-dependent variations in maximum ultrasonic amplitude and propagation velocity, the feasibility of using ultrasound for temperature estimation is investigated. Furthermore, nonlinear ultrasonic techniques are employed to evaluate the detection of early-stage defects, including micro-cracks generated during operation or in the manufacturing process. Tensile fatigue tests were performed in increments of 50,000 cycles up to 200,000 cycles, and results showed that nonlinearity increased significantly at elevated temperatures compared with un-fatigued specimens. Overall, this study demonstrates the potential of ultrasonic techniques for simultaneously measuring temperature and detecting defects in aluminium components, providing a cost-effective alternative to thermistors and improving the reliability of EV battery and motor systems [15,16,17,18,19,20,21].

## 2. Theory

### 2.1. Ultrasonic Theory

Ultrasonic waves are generally classified into two fundamental modes: longitudinal waves, in which particle motion occurs parallel to the direction of wave propagation, and transverse waves, in which particle motion is perpendicular to the propagation direction. Longitudinal waves exhibit the highest acoustic velocity and are capable of propagating through solids, liquids, and gases. In contrast, transverse waves cannot propagate in liquids or gases due to insufficient shear rigidity arising from weak intermolecular cohesion.

(1) $$C_{ls}=\sqrt{\frac{E}{\rho }\frac{1-v}{\left(1+v)(1-2v\right)}}$$

(2) $$C_{ss}=\sqrt{\frac{G}{\rho }}$$

The propagation velocities of longitudinal $C_{ls}$ and transverse waves $C_{ss}$ in a solid medium are described by Equations (1) and (2), respectively, where *E* denotes the longitudinal elastic modulus, *G* the shear modulus, and *v* the Poisson’s ratio. These relationships indicate that ultrasonic wave velocity is dictated by the intrinsic mechanical properties of the material. For metallic materials such as aluminium and steel, changes in density with temperature are minimal; however, elastic moduli decrease significantly as temperature increases. Consequently, ultrasonic propagation velocity decreases with increasing temperature [22,23].

### 2.2. Nonlinear Ultrasonic Theory

Nonlinear ultrasonic waves exhibit the generation of higher-order harmonics when an acoustic wave propagates through a material whose microstructure has been altered by fatigue, plastic deformation, or other forms of damage. This harmonic generation arises from increases in higher-order elastic constants, which cause the stress–strain relationship to deviate from linearity. Thus, while linear ultrasonic theory is insufficient for characterizing materials whose behavior departs from ideal elasticity, nonlinear ultrasonic theory provides greater sensitivity to microstructural changes.

Nonlinearity can generally be categorized into two types: material nonlinearity and geometric nonlinearity. Material nonlinearity originates from nonuniformities within the crystalline lattice, such as dislocations, voids, or precipitates, which induce deviations from ideal elastic behavior. Geometric nonlinearity, on the other hand, occurs when large deformations cause the strain–displacement relationship itself to become nonlinear.

The nonlinear ultrasonic wave equation, shown in Equation (3) [24,25], introduces the third-order elastic modulus as a key governing parameter.

(3) $$u_{tt}-c_{l}^{2}u_{aa}=\left({3c}_{l}^{2}+\frac{C_{111}}{\rho }\right)u_{a}u_{aa}+\left(c_{l}^{2}+\frac{C_{166}}{\rho }\right)\left(v_{a}v_{aa}+w_{a}w_{aa}\right)\;v_{tt}-c_{s}^{2}v_{aa}=\left(c_{l}^{2}+\frac{C_{166}}{\rho }\right)\left(u_{a}v_{aa}+v_{a}w_{aa}\right)\;w_{tt}-c_{s}^{2}w_{aa}=\left(c_{l}^{2}+\frac{C_{166}}{\rho }\right)\left(u_{a}w_{aa}+w_{a}u_{aa}\right)$$

Using perturbation methods, the solution can be expressed as a sum of the fundamental frequency component and the second harmonic component, as shown in Equation (4).

(4) $$u_{tt}^{\left(1\right)}-c_{l}^{2}u_{aa}^{\left(1\right)}=0\;v_{tt}^{\left(1\right)}-c_{s}^{2}v_{aa}^{\left(1\right)}=0\;w_{tt}^{\left(1\right)}-c_{s}^{2}w_{aa}^{\left(1\right)}=0$$

Considering only the longitudinal component, the amplitudes of the fundamental and second harmonic waves are represented by Equation (5).

(5) $$u_{tt}^{\left(2\right)}-c_{l}^{2}u_{aa}^{\left(2\right)}=\left(3c_{l}^{2}+\frac{C_{111}}{\rho }\right)=u_{a}^{\left(1\right)}u_{aa}^{\left(1\right)}$$

In the above equation,

(6) $$A_{2}=\frac{\beta_{l}A_{1}^{2}k_{l}^{2}a}{{8C}_{l}^{2}}$$

The longitudinal second-harmonic nonlinear parameter *β* is defined as the ratio of the second harmonic amplitude to the square of the fundamental amplitude, as expressed in Equation (7). Here, *A* represents the wave propagation distance and f is a frequency-independent function. The relative acoustic nonlinearity parameter, used in this study, is calculated as the ratio of the measured second harmonic amplitude to the square of the measured fundamental amplitude. This normalized parameter provides a quantitative measure of acoustic nonlinearity and is widely used for evaluating material degradation [26,27].

(7) $$\beta^{’}=\frac{A_{2}}{A_{1}^{2}}\propto \beta a$$

### 2.3. Temperature-Dependent Elastic Modulus Theory

It is well established that the elastic modulus of metallic materials decreases as temperature increases. For moderate temperature ranges, this relationship can be approximated using a linear model, as expressed in Equation (8) [28],

(8) $$E\left(T\right)=E_{0}[1-\alpha \left(T-T_{0}\right)]$$

where *E*(*T*) denotes the elastic modulus at temperature *T*, *E_0_* is the modulus at room temperature (20 °C), and *α* is an empirical coefficient representing the temperature dependent rate of modulus reduction. In higher temperature regimes, the temperature dependence of the elastic modulus becomes nonlinear and is better represented by the extended formulation shown in Equation (9). The reduction in elastic modulus with increasing temperature arises from thermally induced lattice vibrations, which weaken interatomic bonding and increase atomic spacing. As a result, the stiffness of the material decreases progressively.

(9) $$E\left(T\right)={E_{0}e}^{-k(T-T_{0})}$$

The nonlinear ultrasonic parameter *β* can be expressed in terms of the second and third-order elastic moduli, *A* and *B*, as shown in Equation (10). Because these elastic constants vary with temperature, β also changes accordingly. As temperature rises, increased lattice anharmonicity intensifies nonlinear elastic effects, generally resulting in increased values of β. This phenomenon is associated with enhanced atomic vibration amplitudes and reductions in higher-order elastic constants, which collectively contribute to stronger nonlinear responses [29,30,31,32,33].

(10) $$\beta =\frac{3B+A}{2+\frac{A}{B}}$$

## 3. Experimental Set Up

**Figure 1:**
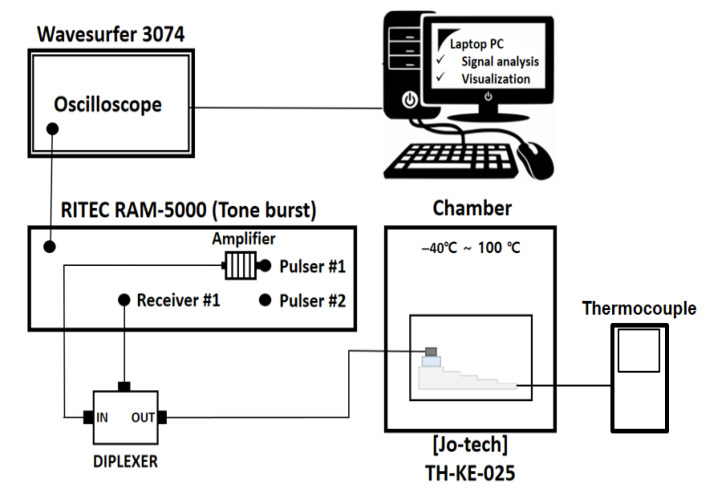
Schematic illustration of the experimental setup used for ultrasonic and nonlinear ultrasonic measurements.

The experimental configuration was organized as shown in Figure 1. In this study, a high-power signal generator (RAM-5000 SNAP, RITEC, Warwick, RI, USA) capable of generating a constant sine wave was used and data were stored with an oscilloscope (Wavsufer 3074, LeCroy crop, NY, USA) for ultrasonic signal collection. A piezoelectric transducer (D790-SM, 5 MHz, Olympus, USA) dedicated to low temperature and high temperature was used, and the temperature was adjusted using a temperature and humidity chamber (TH-KE-025, JEIO TECH, Republic of Korea) to measure the temperature under extreme environmental conditions.

The experiment was conducted by attaching a thermocouple (FSIC-10199, DAIHAN, Republic of Korea) to measure the temperature inside the specimen after it reached the temperature in the chamber. For the fatigue test, a tensile fatigue tester (DTF-505, IDTNT, Republic of Korea) was used.

**Figure 2:**
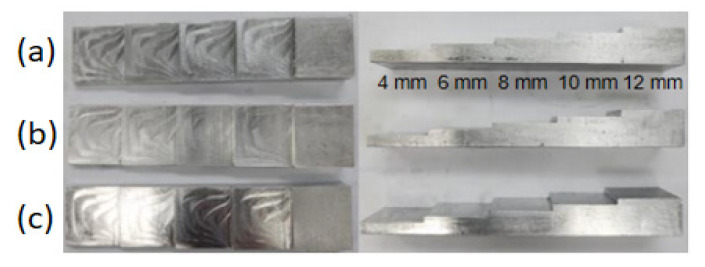
Geometry of aluminium alloy specimens fabricated in a step-wedge configuration: (**a**) Al6061, (**b**) Al6063, (**c**) Al3003.

Figure 2 shows the specimen of each aluminium alloy in a step-wedge configuration. Table 1 summarizes the fundamental mechanical properties of the three aluminium alloys investigated: Al3003, Al6061, and Al6063. Although their alloying compositions differ, all three materials exhibit comparable density and Poisson’s ratio values. However, the tensile strength varies significantly among them, with Al6061 showing the highest strength, followed by Al6063 and Al3003. These differences in mechanical performance are closely related to the distinct alloying elements and heat-treatment characteristics associated with each alloy series. Despite these variations, their room-temperature ultrasonic propagation behavior remains similar due to their comparable elastic modulus values.

Three aluminium alloys commonly used in automotive applications Al6061, Al6063, and Al3003 were selected for evaluation. Step-wedge specimens with thicknesses of 4, 6, 8, 10, and 12 mm were fabricated to examine the influence of thickness on ultrasonic propagation behavior. Although these alloys differ in chemical composition and mechanical properties, their geometric configurations were standardized to ensure consistent comparison across materials. The dimensions and machining processes were carefully controlled to minimize fabrication-induced variability. Since the battery pack and motor have operational efficiency and problems above 70 °C, the maximum amplitude and ultrasonic velocity of ultrasonic waves were measured by increasing the temperature by 20 °C from −40 °C to 80 °C in consideration of abnormal climate.

**Figure 3:**
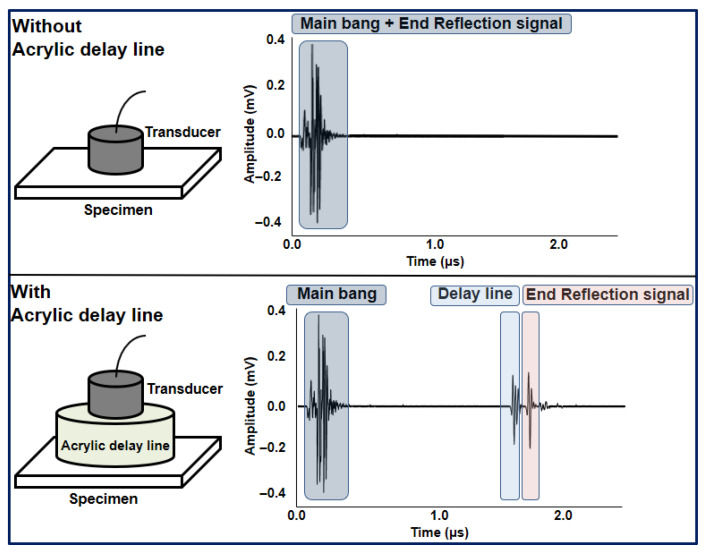
Separation of near-field and reflected ultrasonic signals using an acrylic delay line.

To prevent overlap between the initial excitation (main bang) and the first back-wall reflection, an acrylic delay line was utilized as shown in Figure 3. This delay line increases the effective propagation path before the wave enters the aluminium specimen, ensuring that the near-field region of the transducer does not interfere with the primary reflection signal. As a result, the reflected ultrasonic wave can be clearly distinguished from the initial excitation pulse, allowing more accurate measurement of attenuation, amplitude, and propagation velocity across different temperature conditions.

**Figure 4:**
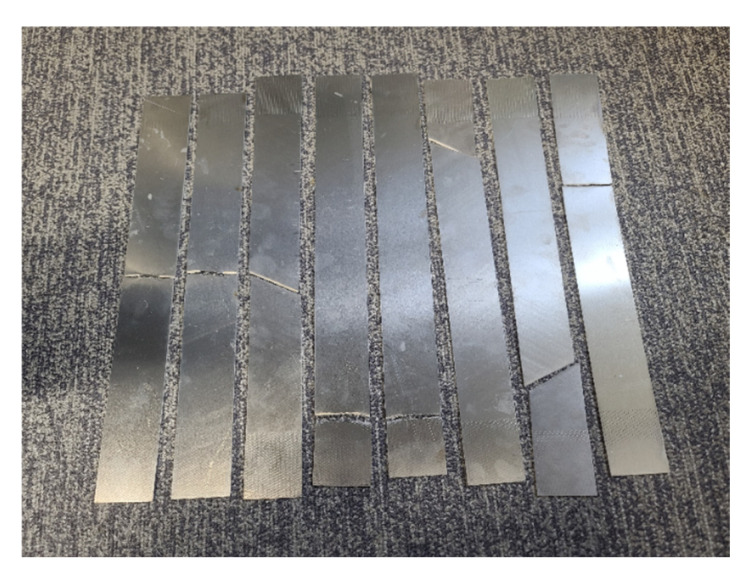
Fatigue test specimens prepared under various tensile loading conditions.

To investigate fatigue-induced microstructural changes and their influence on nonlinear ultrasonic behavior, aluminium plate specimens measuring 400 mm in width, 50 mm in length, and 1 mm in thickness were fabricated, as shown in Figure 4. Tensile fatigue tests were conducted under multiple loading conditions to determine the cycle range in which fracture occurs. Under a maximum load of 10 kN, failure was observed between approximately 220,000 and 250,000 cycles. The detailed fatigue test parameters were as follows: maximum tensile load of 10 kN, minimum load of 1 kN, loading frequency of 5 Hz, stress ratio of 0.1, and a nominal gauge area of 100 mm^2^. Based on these conditions, five sets of specimens were prepared, corresponding to 0, 50,000, 100,000, 150,000, and 200,000 fatigue cycles.

These specimens were subsequently used to evaluate temperature-dependent nonlinearity variations and to assess the sensitivity of nonlinear ultrasonic measurements to accumulated fatigue damage.

All experiments conducted in this study were measured five times, and all results were displayed using error bars.

## 4. Experimental Results

### 4.1. Ultrasonic Result

The ultrasonic pulse signal progressively loses energy as it undergoes repeated reflections, beginning with the initial excitation pulse (main bang). As the reflection sequence continues, each successive echo exhibits increased attenuation due to energy dissipation within the material. In this study, attenuation characteristics at various temperatures were evaluated using the amplitude of the ultrasonic waveform and the first back-wall echo for each thickness of the aluminium alloy specimens.

**Figure 5:**
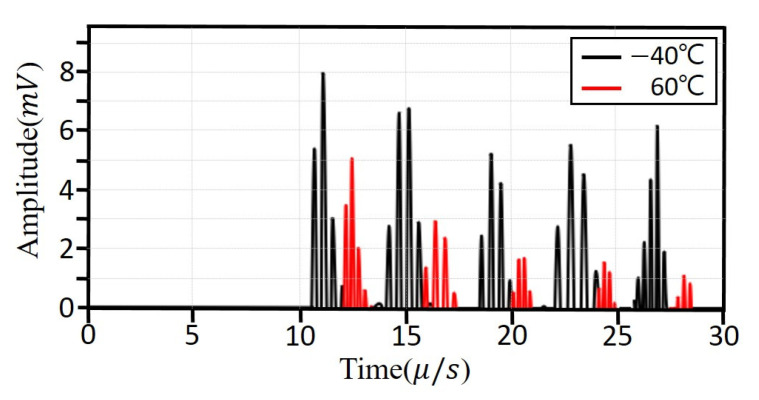
Ultrasonic waveforms of Al6063 measured at −40 °C and 60 °C shown on a common time axis for comparison.

Figure 5 presents the ultrasonic signals of the Al6063 alloy measured at −40 °C and 60 °C, displayed on the same time axis for direct comparison. As temperature increases, signal attenuation becomes more pronounced, and the peak amplitudes of successive reflections decrease accordingly. Additionally, the duration to complete energy decay is shortened at elevated temperatures. To quantitatively compare attenuation, the amplitude of the first reflection—where energy loss is minimal—was used as the reference across all specimens and temperature conditions.

Table 2 summarizes the maximum amplitudes of the first back-wall echo measured for each aluminium alloy across all temperature conditions. Although slight variations were observed among the specimens, Al6063 exhibited marginally higher peak amplitudes in the −20 °C to 40 °C range compared with Al6061 and Al3003. These differences are attributed to variations in alloy composition and corresponding mechanical properties. The experimental results confirm that ultrasonic signal attenuation increases with rising temperature; however, in the low-temperature region (−40 °C to 0 °C), attenuation does not change monotonically. Consequently, it is difficult to establish a temperature dependent linear trend solely from amplitude reduction. This indicates that amplitude based attenuation characteristics alone are insufficient for reliable temperature estimation in aluminium alloys.

Previously, three types of aluminium alloy specimens, Al3003, Al6061, and Al6063, were analyzed for the characteristics of the attenuation of ultrasonic signals according to temperature, and it was confirmed that it was difficult to determine a specific temperature and use it for temperature measurement through the attenuation effect of ultrasonic sig-nals according to temperature. Therefore, in order to promote the ideal possibility for tem-perature measurement of aluminium alloys using ultrasonic testing techniques, we tried to present the ultrasonic propagation velocity in the medium, one of the other ultrasonic propagation characteristics.

**Figure 6:**
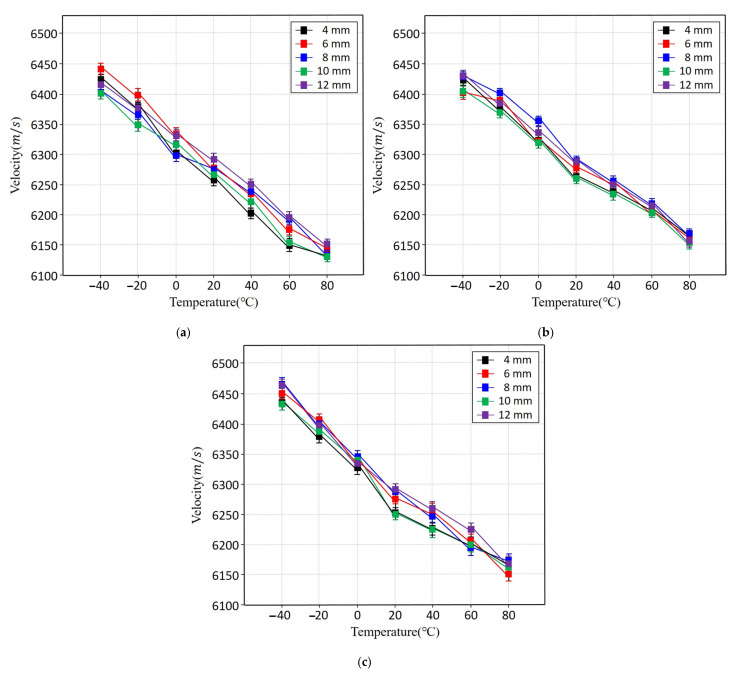
Ultrasonic propagation velocity of aluminium alloys as a function of temperature and specimen thickness: (**a**) Al3003, (**b**) Al6061, and (**c**) Al6063.

Figure 6 and Table 3 present the ultrasonic propagation velocities of the three aluminium alloys over the full temperature range. All alloys exhibited a clear decrease in velocity with increasing temperature, consistent with the reduction in elastic modulus at elevated temperatures. Although Al6061 and Al6063 both belonging to the Al-Mg-Si series showed similar velocity trends, Al6063 demonstrated slightly higher velocities at low temperatures. In contrast, Al3003, an Al-Mn-based alloy, maintained lower velocities in the −40 °C to 0 °C range, reflecting differences in alloy composition and mechanical behavior. At approximately 20 °C, the propagation velocities of all three alloys converged, but at temperatures above 40 °C, the rate of velocity reduction increased. This temperature-dependent decline is attributed to the pronounced decrease in elastic modulus with thermal activation of the lattice. Overall, the experimental data show a nearly linear velocity decrease approximately −47 m/s per °C indicating that ultrasonic velocity is a more reliable indicator for temperature estimation than amplitude-based attenuation.

### 4.2. Nonlinear Ultrasonic Results

**Figure 7:**
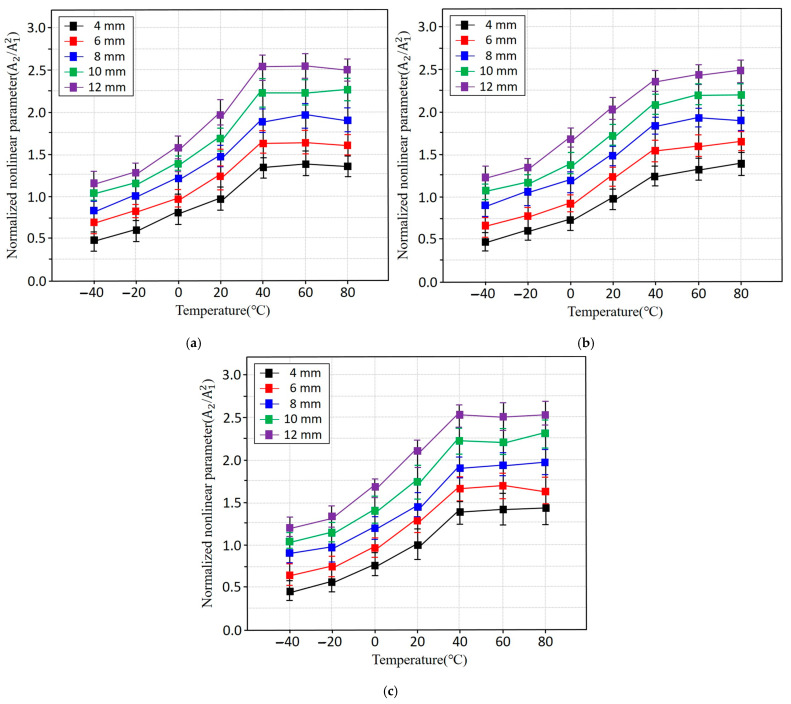
Nonlinear ultrasonic parameter β of aluminium alloys plotted as functions of temperature and specimen thickness: (**a**) Al3003, (**b**) Al6061, and (**c**) Al6063.

Figure 7 illustrates the nonlinear ultrasonic parameters measured as functions of temperature and specimen thickness for all three aluminium alloys. In general, the nonlinearity parameter *β* increases with increasing temperature and thickness. This rise in nonlinearity is attributed to enhanced lattice anharmonicity at elevated temperatures, which increases asymmetry in interatomic potentials. As temperature increases, thermal activation promotes microstructural motions such as the vibration of dislocations, movement of impurities, and local rearrangements of the crystal lattice, thereby strengthening the nonlinear elastic response. The observed increase in β can also be explained by the temperature dependence of higher-order elastic constants. While the second-order elastic modulus decreases gradually with temperature, the third-order modulus decreases more rapidly, resulting in an overall increase in β. This behavior aligns with theoretical predictions for anharmonic solids, where elevated temperatures amplify nonlinear wave matter interactions. The trend is consistent across all three alloys, indicating that alloy composition has only a minor influence on temperature-induced nonlinear behavior.

**Figure 8:**
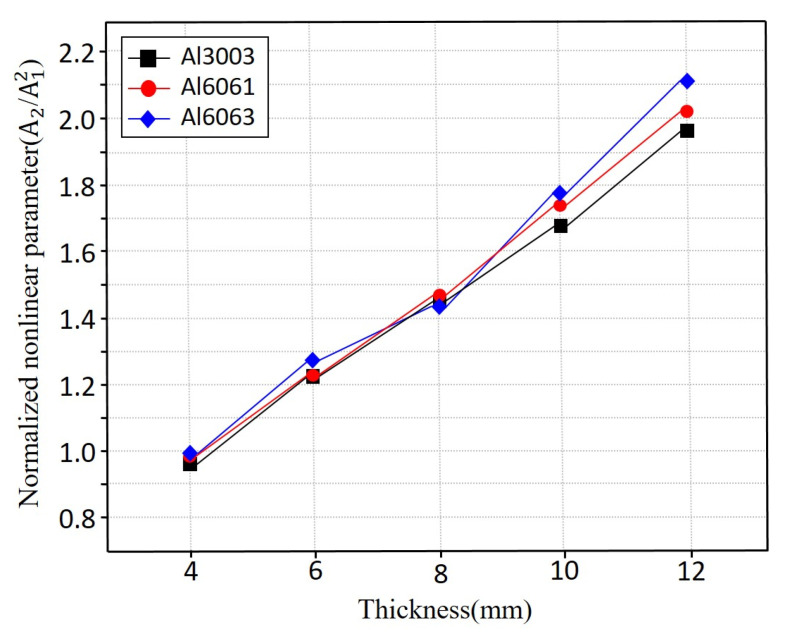
Thickness-dependent nonlinear parameter *β* of aluminium alloys measured at room temperature (20 °C).

As shown in Figure 8, all three aluminium alloys Al3003, Al6061, and Al6063 exhibit similar β values at room temperature across the full range of specimen thicknesses. This indicates that alloy composition has minimal influence on nonlinear ultrasonic response under defect-free conditions. The similarity among alloys also corroborates the ultrasonic velocity trends, which likewise showed only minor differences among the materials. These results suggest that, at room temperature, nonlinearity is governed predominant.

Table 4 shows that the elastic modulus (*E*), shear modulus (*G*), and bulk modulus (*K*) all decrease progressively with increasing temperature. Although the rate of reduction diminishes above approximately 40 °C, the overall decline in stiffness reflects thermally activated lattice vibrations, which weaken interatomic bonding and reduce resistance to deformation. This temperature-induced softening increases the material’s anharmonic response, thereby contributing to a rise in the nonlinear parameter *β*. In many crystalline solids, higher-order elastic constants decrease more rapidly with temperature than second-order constants. As a result, β becomes increasingly sensitive to thermal excitation. For aluminium alloys, the measured increase in β aligns well with established nonlinear elastic theory, which predicts stronger second-harmonic generation as temperature elevates due to enhanced lattice asymmetry and reduced elastic rigidity.

The temperature-dependent nonlinear parameter β can be further interpreted using Equation (11),

(11) $$B\left(T\right)=B_{0}[1+k\left(T-T_{0}\right)]$$

where *β*_0_ represents the reference value at temperature *T*_0_ and *K* is the temperature coefficient. For metallic materials, *K* typically ranges between 0.002 and 0.003, and for aluminium alloys, a value of approximately 0.0025 is widely accepted [34]. Using this coefficient, *β* at elevated temperatures can be predicted from room temperature measurements, and the resulting trend aligns well. Although the same *β* trend was observed for all three alloys, deviations at higher temperatures may result from external experimental factors rather than intrinsic material behavior. The transducer used in this work (D790-SM, 5 MHz, Evident, Quebec City, Quebec) is rated for temperatures between −20 °C and 500 °C; however, cable performance, thermal gradients within the specimen, and insufficient thermal stabilization time can influence measurements. For improved accuracy, specimens should be held at the target temperature for extended periods to ensure full thermal equilibrium [35]. These considerations are essential when interpreting *β* measurements collected under short-duration high-temperature conditions.

### 4.3. Nonlinear Ultrasonic Result for Fatigue Test Specimens

**Figure 9:**
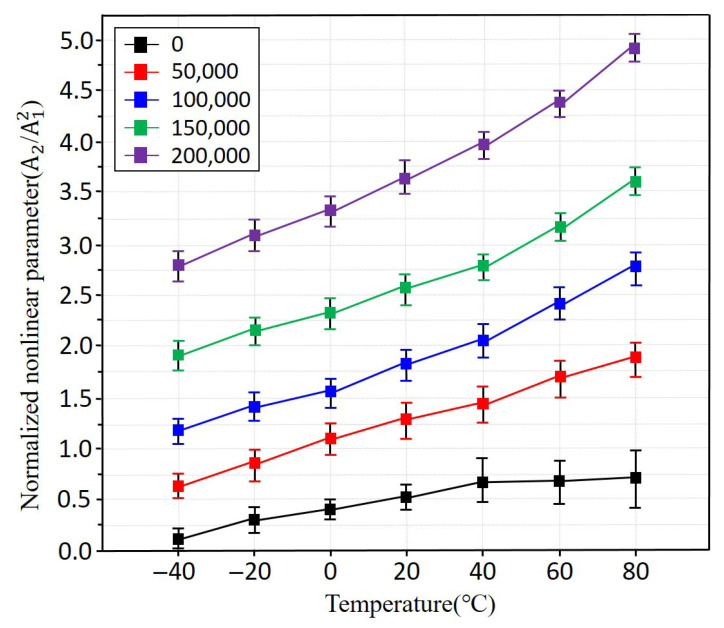
Temperature-dependent nonlinear parameter β measured for fatigue-damaged specimens subjected to different fatigue cycle levels.

Figure 9 presents the nonlinear ultrasonic parameter *β* measured for fatigue-damaged specimens subjected to different numbers of tensile loading cycles. In general, it is well known that the nonlinearity parameter increases with increasing fatigue [36,37]. However, for automotive components operating at short-term temperatures, accurate temperature-dependent nonlinearity measurements are not possible. Therefore, it is crucial to determine whether the effects of defects or material micro-changes can be identified even in short-term conditions. As shown in Figure 7, the non-fatigued specimens do not exhibit an increase in nonlinearity above 40 °C due to insufficient material degradation. However, as fatigue accumulation progresses from 50,000 to 200,000 cycles, β increases steadily, indicating enhanced microstructural degradation such as dislocation accumulation, slip band formation, and early-stage microcracking. Temperature effects further amplify this trend. At elevated temperatures, increased lattice anharmonicity enhances sensitivity to microstructural damage, resulting in more pronounced nonlinear responses. Conversely, at lower temperatures, β exhibits reduced sensitivity, reflecting the suppression of thermally activated lattice motion. Overall, the combined influence of fatigue damage and temperature demonstrates that nonlinear ultrasonic techniques are capable of detecting early-stage degradation in aluminium alloys, even before observable macroscopic defects appear.

## 5. Conclusions

This study comprehensively investigated the temperature-dependent ultrasonic and nonlinear ultrasonic characteristics of aluminium alloys widely used in electric vehicle components, including battery housings, structural frames, and motor assemblies. Three alloys—Al3003, Al6061, and Al6063—were evaluated through a combination of conventional ultrasonic measurements and nonlinear ultrasonic techniques, and the effects of temperature, thickness, and fatigue-induced microstructural degradation were systematically analyzed.

First, the ultrasonic attenuation of all three aluminium alloys increased as the temperature rose, consistent with enhanced internal friction and thermally activated atomic motion. Although attenuation generally increased, the low-temperature region (−40 °C to 0 °C) exhibited non-monotonic behavior, indicating that amplitude-based attenuation alone is insufficient for reliable temperature estimation.

Second, ultrasonic propagation velocity demonstrated a highly linear and repeatable dependence on temperature across all alloys and thicknesses. A linear relationship with a slope of approximately −47 °C was confirmed, reflecting the substantial reduction in elastic modulus at elevated temperatures. This strong linearity suggests that ultrasonic velocity can serve as a robust alternative to conventional thermistors, enabling nondestructive, real-time temperature estimation in aluminium structures without the need for embedded contact sensors.

Third, nonlinear ultrasonic measurements revealed that the nonlinear parameter *β* increased with both temperature and specimen thickness. This behavior is attributed to the temperature-dependent softening of higher-order elastic constants and the corresponding enhancement of lattice anharmonicity.

Fourth, tensile fatigue experiments confirmed that *β* is highly sensitive to microstructural degradation. Specimens subjected to 50,000 to 200,000 fatigue cycles exhibited a progressive increase in *β*, corresponding to the accumulation of dislocations, slip bands, and early-stage microcracking. Temperature effects further magnified *β*, with elevated temperatures enhancing sensitivity to fatigue-induced defects.

Overall, the combined experimental results demonstrate that ultrasonic velocity offers a highly linear and reliable indicator for temperature estimation, whereas the nonlinear parameter *β* provides excellent sensitivity to early fatigue damage. Therefore, ultrasonic techniques, particularly those combining linear and nonlinear measurements, offer a promising nondestructive alternative to thermistors for temperature monitoring and structural health assessment in aluminium components used in electric vehicles.

## Figures and Tables

**Table 1:** Mechanical properties of aluminium alloys.

Material	Tensil Strength (MPa)	Density (g/cc)	Young’s Modulus	Poisson’s Ratio
Al3003	150	2.8	69	0.33
Al6061	310	2.7	69	0.33
Al6063	240	2.7	69	0.33

**Table 2:** Peak amplitude of the first back-wall echo for each aluminium alloy as a function of temperature.

Average for Peak Amplitude of 1st Echo (mV)							
Material	Temperature (°C)						
	−40	−20	0	20	40	60	80
Al3003	6.74	6.51	6.28	5.25	3.90	1.63	0.56
Al6061	6.20	5.75	5.75	4.64	2.87	1.12	0.39
Al6063	6.66	5.87	5.74	4.23	2.43	1.41	0.40

**Table 3:** Ultrasonic wave propagation velocity of aluminium alloys as a function of temperature and specimen thickness (*m*/*s*).

Average Ultrasonic Velocity (*m*/*s*)							
Material	Temperature (°C)						
	−40	−20	0	20	40	60	80
Al3003	6418	6370	6316	6272	6229	6170	6140
Al6061	6419	6380	6328	6278	6248	6207	6170
Al6063	6453	6397	6333	6272	6246	6208	6169

**Table 4:** Temperature-dependent reduction in elastic modulus for aluminium alloys.

Elastic Modulus	Temperature (°C)						
	−40	−20	0	20	40	60	80
*E*(*T*)	71.68	71.12	70.56	70.00	69.44	68.88	68.32
*G*(*T*)	26.95	26.75	26.54	26.32	26.11	25.90	25.69
*K*(*T*)	70.24	69.69	69.15	68.60	68.05	67.51	66.96

## Data Availability

The data supporting the findings of this study are not publicly available due to security restrictions of the providing company. However, the data may be made available upon reasonable request and subject to review.
